# Brazilian Agroindustrial Wastes as a Potential Resource of Bioative Compounds and Their Antimicrobial and Antioxidant Activities

**DOI:** 10.3390/molecules27206876

**Published:** 2022-10-13

**Authors:** Alaor Valério Filho, Luisa Bataglin Avila, Douglas Hardt Lacorte, Thamiris Renata Martiny, Vanessa Rosseto, Caroline Costa Moraes, Guilherme Luiz Dotto, Neftali Lenin Villarreal Carreno, Gabriela Silveira da Rosa

**Affiliations:** 1 Graduate Program in Materials Science and Engineering, Technology Development Center, Federal University of Pelotas, Pelotas 96010-610, Brazil; 2 Chemical Engineering Department, Federal University of Santa Maria, Santa Maria 97105-900, Brazil; 3 Graduate Program in Materials Science and Engineering, Federal University of Pampa, Bagé 96413-172, Brazil; 4 Chemical Engineering, Federal University of Pampa, Bagé 96413-172, Brazil; 5 Food Engineering, Federal University of Pampa, Bagé 96413-172, Brazil

**Keywords:** olive leaves, jaboticaba peel, araçá peel, pecan nut shell

## Abstract

The study of the recovery of bioactive compounds from natural resources and its implications in several areas is very significant for the scientific community. This work aimed to study Brazilian agroindustrial wastes’ antioxidant and antimicrobial activities using green extraction. Olive leaves, jaboticaba peel, araçá peel, and pecan nut shells were evaluated under four conditions: (1) convective-drying and aqueous extraction, (2) convective-drying and ethanolic extraction, (3) freeze-drying and aqueous extraction, and (4) freeze-drying and ethanolic extraction. The results demonstrated that all samples showed high antioxidant potential, and the highest antioxidant activity was obtained for the extract of pecan nut shell. As for the quantification of compounds by HPLC, the olive leaf presented the highest content of phenolic compounds in the extract, mainly oleuropein. Finally, the antimicrobial activity analysis revealed the extracts’ bactericidal potential against *Staphylococcus aureus* and *Escherichia coli*. The present study shows that green extraction can extract bioactive compounds with antioxidant and antimicrobial properties, highlighting the importance of choosing the drying method and solvent for future uses of these natural resources by the industry.

## 1. Introduction

Over the years, knowledge about the chemical nature of various bioactive compounds and the effect on human health caused by synthetic additives has been widely disseminated. Thus, this fact prompted several industries, such as pharmaceuticals, pesticides, and food, to search for natural sources of active compounds [1,2,3]. The bioactive compounds, such as fruits, peel, leaves, and roots, can be abundant in nature and show beneficial effects on human health [4]. Among these benefits are anticarcinogenic, anti-inflammatory, cardioprotective, neuroprotective, antioxidant, antimicrobial, hepatoprotective, antidiabetic, and other actions [4,5,6,7]. In this sense, an alternative is the use of agro-industrial residues that are known as rich sources of bioactive compounds. Furthermore, these byproducts are generated on a large scale, which makes them interesting from an economic point of view, in addition to being sustainable [4,5,7,8,9,10].

Brazil stands out for harboring an important environmental asset [11]. With six different biomes, it has a great diversity of products cultivated in each region. For example, in the Rio Grande do Sul, two biomes are located, the Pampa and the Atlantic Forest. Since colonization, the southern region of the state, where the Pampa biome is located, has been characterized by high agricultural activity due to the poverty of the population at the time and the possibility of extracting natural resources in this region [11,12]. Among the different types of agro-industrial wastes in this region, olive leaves, jaboticaba peel, pecan nut shells (lignocellulosic endocarp), and araçá peel can be mentioned.

Olive leaves are generated in large volumes during pruning and olive harvesting. The interest in using olive leaves is due to the high content of phenolic compounds, such as flavonoids, and secoiridoids, especially oleuropein [13,14,15]. Mohamed et al. [16] investigated the olive mill wastewater, and pointed out that the remaining phenolic compounds have potential for bacterial inhibition and antioxidant activity. On the other hand, jaboticaba and araçá peel constitutes the byproducts of the food industry that process the fruits to produce juices, jellies, and liquors [17,18]. In jaboticaba and araçá, the bioactive compounds are present mainly in its peel, with cyanidin-3-glucoside as the main anthocyanin found [18,19]. The interest in pecan nut shells is the same, the presence of phytochemicals, with special emphasis on the shells that concentrate the greatest number of polyphenols. Besides that, their extracts show a high antioxidant and antimicrobial potential [20,21,22,23].

The interest in using these residues is mainly due to their antioxidant and antimicrobial potential. Thus, once the bioactive compounds are extracted, they can be used as natural additives. Although the present compounds are of great interest, their effective use requires some pre-treatments of the raw materials [1,15,18,23,24,25]. An example of the pre-treatment is drying, which can improve postharvest life relatively low in fresh plant matrices. The most common technique used to dry plant materials is convective-drying. However, high temperatures can degrade the bioactive compounds [26]. Therefore, freeze-drying is an alternative for dehydrating more sensitive bioactive compounds. First, a negative temperature is used to freeze the sample, and after, it is subjected to low pressure. At this stage, the sublimation of the formed ice crystals occurs. However, this drying technique has a high energy cost due to the need for a vacuum pump during the entire process [27]. For this reason, studying drying conditions and methods is very important [28,29,30]. Moreover, the choice of solvent type is important in the extraction technique. In this sense, green solvents have stood out, as they have a less environmental impact and fewer restrictions on safety in some applications and can be used in the food and pharmaceutical industries, for example [31,32]. Therefore, knowing the nature of the compounds that will be extracted is an important strategy for improving the extraction [33,34].

In this scenario, the present work aimed to evaluate the composition of four plant matrices extracts (olive leaves, jaboticaba peel, red araçá peel, and pecan nut shells), and considered Brazilian agro-industrial wastes. In this regard, the effect of the drying process (convective-drying and freeze-drying) of the raw materials on the final composition of the extract was evaluated, as well as the type of solvent used during the green extraction.

## 2. Results and Discussion

### 2.1. Antioxidant Activity

DPPH radical scavenging capacity (RSC_DPPH_) results for all extracts in which the different preparation conditions were evaluated are summarized in Table 1. Extracts obtained from freeze-drying biomass using distilled water and ethanol 40% as extracting solvent were named FW and FE, respectively. CW and CE refer to extracts produced with convective-dried biomass, using water and ethanol 40%, respectively.

All extracts tested showed excellent results for the RSC_DPPH_, ranging from 57.77% to 95.23%. For the jaboticaba peel extracts, the only treatment that showed a significant difference (*p* < 0.05) was the CW, in which the extraction with water reduces the antioxidant capacity of the jaboticaba peel. The highest percentage of RSC_DPPH_ among all extracts was observed for olive leaf extract, not significantly differing (*p* < 0.05) for all treatments. The extraction with water also impaired the RSC_DPPH_ of the araçá peel, with no significant difference in different drying techniques. Finally, extraction with water and the freeze-drying process (FW) for pecan nut shell extracts were less efficient, resulting in a lower RSC_DPPH_. Ethanol solvent favored the RSC_DPPH_ of the extracts (Table 1). Different drying techniques did not significantly impact the results when comparing only the use of ethanol.

Nobossé et al. [35] evaluated the influence of extracting solvent (water, ethanol, and methanol) on the production of *Moringa oleifera* L. leaf extract and obtained higher values of RSC_DPPH_ for ethanolic extract of 53.3–71.1%, this variation is related to the time of storage of samples after collection, which ranged from 30 to 60 days. Meira et al. [36] obtained the RSC_DPPH_ value for jaboticaba peel extract of 86.31%, using a 1:2 water:ethanol solution as extracting solvent. Pitz et al. [37] obtained RSC_DPPH_ of 91.01% for jaboticaba peel extract using microwave-assisted extraction and 50% (v/v) ethanolic solution as extracting solvent. Martiny et al. [33] and Rosa et al. [27] obtained values of 93.58% and 90.03% for olive leaf extract obtained from microwave-assisted extraction.

Salvador et al. [38] analyzed the RSC_DPPH_ of the pecan nut oil extraction residue and obtained the best result of 79% for ultrasound-assisted extraction using ethanol as extracting solvent. Meregalli et al. [39] reached the value of 86.31% for RSC_DPPH_ for araçá extract using an ultrasound-assisted extraction method and petroleum ether as extracting solvent.

Table 2 shows the results of the extracts that were also evaluated for antioxidant activity according to the ferric reduction.

For jaboticaba peel extracts, the absolute best result was for the FE treatment. For the other extracts, the best treatment was CE. However, it is noteworthy that the pecan nut shell extract presented higher FRC. In addition, the best condition for the extracts from pecan nut shells was obtained for the freeze-drying sample using 40% of ethanolic solution as extracting solvent, not significantly differing (*p* < 0.05) from the CE and FW extracts.

Similar results to those found for the natural extracts of this study have already been reported in the literature. Rufino et al. [40] analyzed methanolic extracts from the pulp and peel of jaboticaba and found an FRC of 87.9 µM Fe^+2^ g^−1^. Kashaninejad et al. [41] studied the antioxidant activity of ethanolic extracts of olive leaves from Spain and Iran. They obtained 1254.48 µM Fe^+2^ g^−1^ and 1075.27 µM Fe^+2^ g^−1^, respectively, evidencing the differences in the leaf collection regions. Denardin et al. [42] produced ethanolic extracts from the edible part of araçá and found FRC: 89.09 µmol FeSO_4_ 7H_2_O g^−1^. For Bambara peanut extract, NYAU et al. [43] found FRC values between 801 and 970 µM Fe^+2^ g^−1^, using microwave-assisted extraction and methanol solution 70% (*v*/*v*) as an extracting solvent. In this regard, it is noteworthy that in this study, biomasses are considered waste; even so, they showed competitive values for FRC.

RSC_DPPH_ and FRC assay described the ability of tested samples to neutralize reactive species and modulate redox tone. For this result to be better achieved, ethanol as a solvent is recommended since the drying method did not significantly impact this result in general for the antioxidant activity. The results found in this work are in line with those found in the literature that used the same plant species. The difference between the RSC_DPPH_ and FRC values reported in the literature and the results obtained in this work may be related to several factors, some of which are: biomass species, soil quality, storage time, transport conditions after harvest, drying conditions of the biomass, sample cleaning method, biomass particle size, extraction technique and extracting solvent [44]. However, these results demonstrate that the extraction technique by maceration presented competitive values for RSC_DPPH_ and FRC. Furthermore, it is possible to verify the use of eco-friendly solvents, such as water and ethanol, to obtain an extract with a high percentage of RSC_DPPH_ and FRC from the studied biomasses.

### 2.2. Total Phenolic Compounds

Table 3 shows total phenolic compound results from all extracts evaluated in the present study.

In Table 3, the natural extract that presented the highest amount of TP was the pecan nut shell extract. Furthermore, there was no significant difference between the ethanolic extract, convective-dried, and freeze-dried (*p* < 0.05). On the other hand, the jaboticaba peel extract showed a significant difference in almost all experimental conditions, obtaining the highest value in the FE sample. Furthermore, for the olive leaf extract, the highest value of TP was obtained for the CE sample, and for the araçá peel extract, the highest value was obtained for FE.

Lenquiste et al. [45], for the aqueous extract of jaboticaba peel, obtained the TP of 36.12 mg_GAE_ g^−1^ and 48.61 mg_GAE_ g^−1^ for the methanolic extract 70% (*v*/*v*). In both cases, maceration extraction was used. Martiny et al. [44] obtained a 41.40 mg_GAE_ g^−1^ for olive leaf extract using maceration extraction and water as the extracting solvent. Salvador et al. [46], using ultrasound-assisted extraction and acetone 64% (*v*/*v*) as extracting solvent, obtained the value of 100 mg_GAE_ g^−1^ for the extract of the residue from the extraction of pecan nut oil, and Bittencourt et al. [47] a value of 136.95 mg_GAE_ g^−1^ for araçá peel extract using supercritical extraction and ethanol as extracting solvent. The results of the present work were competitive with those reported in the literature, demonstrating the relevance of studying other techniques and other methods of sample preparation to extract phenolic compounds.

The results obtained for the total phenolic compounds agree with what was previously observed for both RSC_DPPH_ and FRC for antioxidant activity. This fact can be attributed to the phenolic content in the samples, which are known for their antioxidant activity [48,49].

The most favorable drying method for each sample was determined based on the antioxidant activity and total phenolic compounds results (Table 1, Table 2 and Table 3). Freeze-drying was better for the jaboticaba peel, and araçá peel ethanolic extracts, while convective-drying was better for the olive leaves and pecan nut shell ethanolic extracts

Due to differences in the chemical properties of each phenolic compound in different plant species, the ideal extraction solvent choice depends on the plant type and the phenolic compound of interest. Therefore, CE and CW samples were chosen for olive leaf, pecan nut shell extracts, and FE and FW for jaboticaba peel and araçá peel extract to proceed with HPLC analysis.

### 2.3. Phenolic Compounds Analysis by High-Performance Liquid Chromatography—HPLC

The individual phenolic compounds that HPLC could identify have been listed in Table 4 and the chromatograms are shown in Figure S1. In the evaluated extracts, we identified phenolic acids (gallic, cafeic, *p*-coumaric, *trans*-cinnamic, and *trans*-ferulic), flavonoids (quercetin and kaempferol), anthocyanins (cyanidin-3-glucoside), phenolic alcohols (hydroxytyrosol and tyrosol), secoiridoid (oleuropein) and hydroxynamic acid derivative (verbascoside).

In general, the extraction of the analyzed phenolic compounds was favored using the ethanolic solution as extracting solvent. The exception occurred for gallic acid for jaboticaba peel, olive leaves, and pecan nut shells. This same behavior has been reported previously. Shing et al. [50] analyzed the extraction of gallic acid from aryl pomegranate using water, ethanolic solution 80% (*v*/*v*), and the mixture of ethanol:water:ether (8:1:1, *v*/*v*) as extracting solvents. The best results were obtained for ethanol:water:ether, followed by water and, finally, ethanol. This trend may be because the ether modulates ethanol’s polarity, improving hydrolyzable phenolic compounds’ solubility [50].

The jaboticaba peel extract has a high anthocyanin content, as already reported in the literature. This behavior can be seen in Table 4, where the highest amount of phenolic compound was identified for cyanidin-3-glucoside, with a value of 8.83 mg g^−1^. In addition, compared with the study carried out by Andrade Neves et al. [51], the value obtained for jaboticaba peel extract of the same species was 0.25 mg g^−1^, using a water solution, methanol, and formic acid as the extracting solvent. Inada et al. [52] quantified the phenolic profile of different parts of the fruit by HPLC and reported that jaboticaba peel has the highest amount of total phenolic content (2252 mg 100 g^−1^, d.w.). Jaboticaba is associated with many health benefits related to its phenolic composition. Albuquerque et al. [53], reported that jaboticaba peel extract might be a natural anti-inflammatory alternative. These health benefits promoted by the bioactive compounds of jaboticaba resulted in a patent related to the use of the alcoholic extract of jaboticaba peel for the treatment of metabolic processes, such as the healing of injuries caused by aging [54].

Notably, the extract of the araçá peel was also rich in cyanidin-3-glucoside. However, compared with the jaboticaba peel extract, the araçá extract shows a significant difference (*p* < 0.05), with a value of 1.63 mg g^−1^. This work’s results agree with the results obtained by Denardin et al. [38], who also detected gallic acid and quercetin as major compounds for araçá extracts. Few reports in the literature assess the phenolic profile of the araçá. However, hyperoside (flavonoid) was the main phenolic compound in araçá, followed by cyanidin [55].

Despite this, araçá extract still has a higher value than other biomasses reported in the literature, such as blueberry, red pitaya, blackberry, and eggplant, studied by Vieira et al. [56], using 70% ethanol as extracting solvent. The cyanidin-3-glucoside levels were also higher than those presented by Pereira et al. [18] (1.20–1.45 mg g^−1^), who evaluated araçá red genotypes using methanolic extraction but exhibited lower contents than the purified extract (11.6–12.9 mg g^−1^). The antioxidant potential of cyanidin-3-glucoside has been extensively studied in recent years, highlighting its modulating action on the action of related enzymes such as the oxidative stress regulatory factor (Nrf2), DNA protection against UV-B radiation, anti-inflammatory, and cytoprotective action, in addition to in the death of cancer cells [57].

The main phenolic compounds in olive leaf extract were oleuropein, verbas-coside, hydroxytyrosol, and tyrosol, which were expected and have already been reported in the literature [58,59]. However, the levels of oleuropein in the ethanolic extracts (130.45 mg g^−1^) were relatively higher compared to other studies. Ghomari et al. [60] evaluated the olive leaf extract and obtained the value of 80.67 mg g^−1^ of oleuropein, using extraction by two-step maceration, using first ethanol as extracting solvent and then water. This difference may be related to the temperature of 25 °C used in the extraction. Rosa et al. [32] already reported that the increase in temperature favors the extraction of phenolic compounds such as oleuropein from olive leaves. Lama-Muñoz et al. [61] evaluated different extraction methods (Soxhlet and pressurized liquid extraction) for different cultivars obtaining oleuropein levels of 43.4–122.3 mg g^−1^. Oleuropein, hydroxytyrosol, and verbascoside possess ideal chemistry for free radical scavenging, acting as an antioxidant. At the same time, tyrosol has weak antioxidant activity, but it is a very stable compound compared to other polyphenols and less subject to autooxidation [59,62].

For pecan nut shell extract, the extraction and chromatographic conditions allowed only the identification of gallic acid. Prado et al. [63] highlighted the difficulty in separating the components using such biomass, as the authors performed different extraction methods, and only the Sephadex^®^ LH-20 resin was able to remove the components that interfered with the chromatograms, making it possible to identify some phenolic acids, such as gallic acid, and flavonoids. Because of analytical difficulties, few studies on the phenolic composition of pecan nut shells have been published. De La Rosa et al. [21], using HPLC, found only the presence of gallic acid and ellagic acid. However, Hilbig et al. [64] detected a more comprehensive phenolic profile, which they found in addition to gallic acid, catechin, epicatechin, epigallocatechin, and epicatechin gallate. Their results indicated that pecan nut shell extracts are effective against tumor cell growth and may be considered an alternative to cancer treatment.

The phenolic compounds of biomass are determined by genetic and environmental factors and can be modified by oxidative reactions during extraction and storage. In addition, the phenolic composition is metabolized as a defense response against intense solar radiation and other adverse factors [65]. The variations in the studied biomass result from the formation route of these compounds. Thus, the different contributions of individual phenolics in the extracts are expected to produce different antioxidant and antimicrobial effects.

Therefore, optimal recovery of phenolic compounds requires that the solvent be selected based on the plant and the extraction of the phenolic compounds and hence on, antioxidant activity. Although the extraction of antioxidant compounds usually takes place through organic solvents, among which the most common are ethanol and methanol, water is sometimes also adequate [66]. In the present research, the ethanol extracts showed higher or no significant differences than the distilled water extracts. Based on results on the efficiency of ethanol in extracting phenolic compounds and antioxidant activity, and due to the low toxicity of ethanol as recommended by the Food and Drug Administration (FDA), extracts prepared with ethanol were selected for the microbiological analysis.

### 2.4. Microbiological Analysis

**Figure 1:**
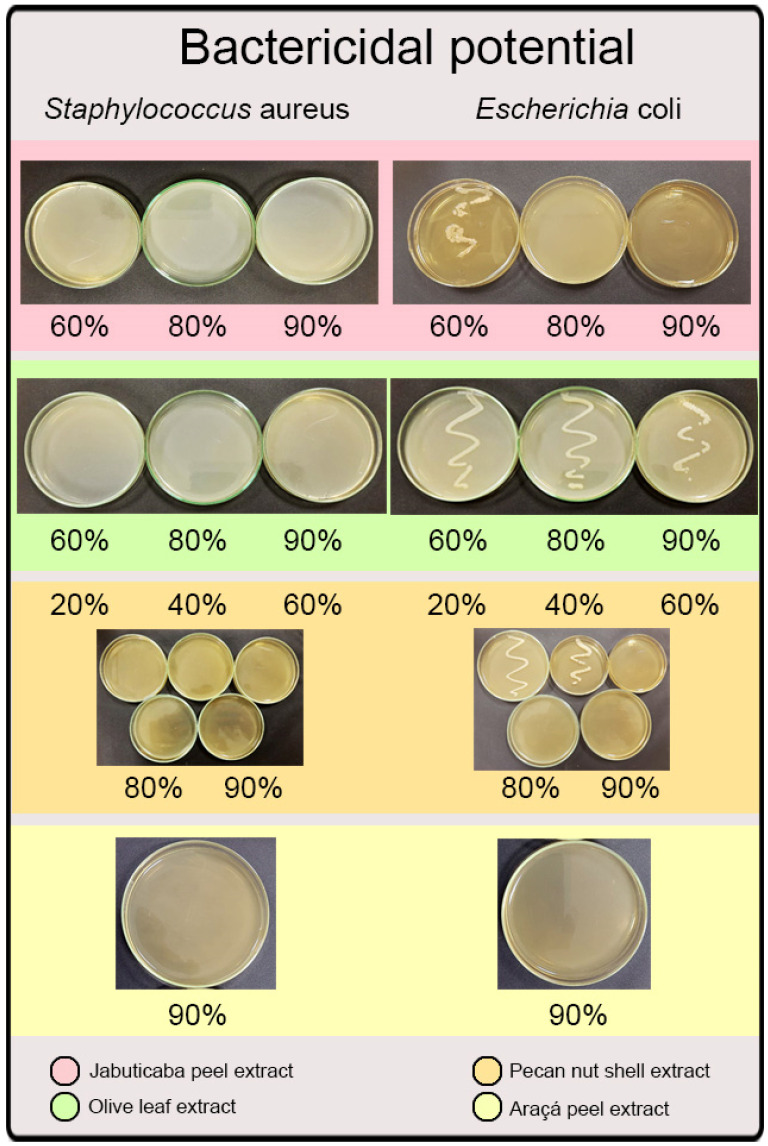
The bactericidal potential of the natural extracts against microorganisms *Escherichia coli* and *Staphylococcus aureus*.

The extraction conditions evaluated promoted a good recovery of the main phenolic compounds for olive leaf extract (oleuropein), jaboticaba peel, and araçá peel extract (cyanidin-3-glycoside). Both phenolic compounds have great bactericidal potential, promoting this characteristic of the extract [56,63]. Although HPLC analysis failed to identify the individual phenolic compounds in the pecan nut shell extract, it is rich in bioactive compounds with potential antimicrobial action. Table 5 shows the inhibition results, and Figure 1 shows the bactericidal potential of the extracts against the microorganisms studied.

Through the inhibition analysis (Table 5), the minimum inhibitory concentration (mic) for the studied extracts was possible. The extract of olive leaf and jaboticaba peel showed m_ic_ at a concentration of 60% (*v*/*v*) for both microorganisms. Due to the extract’s natural turbidity, it was impossible to visualize the inhibition potential for the pecan nut shell extract. The araçá extract sample showed m_ic_ only at a concentration of 90%.

In Figure 1, it is possible to verify the minimum bactericidal concentration of all extracts. The only extract that did not show bactericidal potential at any dilution was that of olive leaf for *Escherichia coli*. According to Gould et al. [67], this can be attributed to the profile of Gram-negative microorganisms since they are more resistant than Gram-positive microorganisms, mainly because of the protective layer of polysaccharides, which makes the action of antimicrobials difficult. However, the pecan nut shell extract, even at the highest dilution (20%, *v*/*v*), showed bactericidal potential for the microorganism *Staphylococcus aureus*. All extracts showed promising results, demonstrating the potential to be applied in different industry sectors as a bactericidal agent.

The polyphenols detected from the extracts of all the studied biomasses, such as flavonoids, may have the ability to penetrate the phospholipid matrix of animal cells due to their hydrophobic characteristic. Thus, the main potential mechanism in its antimicrobial activity is iron chelation, reducing the activity of essential ions, inhibiting cell wall synthesis, and, consequently, rupturing cell membranes [68]. As Gram-negative bacteria present a specific lipid barrier in the cell wall, protecting them against hydrophobic compounds, the antimicrobial effect of jaboticaba peel, olive leaves, and pecan nut shell extracts in Gram-negative bacteria is lower than in Gram-positive [69], as was explicitly found in the results of antimicrobial analysis (Figure 1). Although the results of jaboticaba and araçá extracts are due to the anthocyanins, they can inhibit Gram-negative and Gram-positive bacteria since they react with DNA.

The literature has already reported extracts from these and other biomasses that also had the potential to inhibit the microorganisms *Staphylococcus aureus* and *Escherichia coli*, such as *Vaccinium corymbosum* [70], the leaf of *European chestnut* [71], *Coriolus versicolor* [72], the leaf of *Azadirachta indica* [73], the leaf of *Hypericum roeperianum* and *Cremaspora triflora* [74], the shell of *Carya ilinoinensis* [63,75], the leaf of Olea europaea [44], the peel of *Plinia cauliflora* [26] and the peel of *Psidium cattleianum* Sabine [76].

The antimicrobial activity of the phenolic compounds identified and quantified in the present study has been reported in the literature. Hydroxytyrosol, oleuropein, and verbascoside showed antibacterial activity against *Staphylococcus aureus* [77,78]. Sanhueza et al. [79] evaluated grape extract’s antibacterial effect against Staphylococcus aureus and Escherichia coli. They identified phenolic acids such as gallic acid and *p*-coumaric acid and flavonoids such as quercetin and kaempferol. The authors evaluated the fractional inhibitory concentration index. They found similar values for both compounds in high concentration in the extract and phenolic compounds in low concentration, suggesting that each compound contributes to an integrated action, favoring synergistic action between them and other compounds such as antibiotics.

### 2.5. Main Findings and Future Research Directions

(i) The extracts obtained from agro-industrial residues in the Rio Grande do Sul, Brazil, presented competitive results compared to the literature. This trend is extremely relevant since the Rio Grande do Sul is historically characterized by high harvesting activity and other processes involved in the food industry, which generates a huge volume of waste [11].
(ii) The freeze-drying process has an advantage over the conservation of bioactive compounds from plant samples. However, it is more expensive and time-consuming [27]. Therefore, the superior results using the convective-drying obtained for olive leaf extract and pecan nut shell extract present a better and more economical alternative for producing extracts from these biomasses.
(iii) Despite the promising results regarding the variables extractor solvent (water or ethanol 40%) and drying method (convective-drying and freeze-drying), other variables can be evaluated in future works, such as type of extraction, temperature, and pH.
(iv) Although the pecan nut shell extract showed higher values of TP than the other extracts studied in this work, all extracts have relevant results that allow their application in several areas, such as pharmaceuticals, cosmetics, food packaging, and medicine, among others.
(v) The results obtained in this work, using water or ethanol (40%) as extracting solvent, have great potential for industrial use since there are few restrictions since they are green solvents.
(vi) Byproducts can be used to complement or create new products with health and technological benefits through application in the food, pharmaceutical, and cosmetic industries. For example, processing jaboticaba peel, olives leaves, araçá peel, and pecan nut shells takes advantage of these raw materials to develop innovative and healthier products. In addition, using byproducts can promote more efficient use of natural resources. However, specific in vitro, in vivo, and clinical trials must be performed to confirm the benefits to human health or attest to these biomasses as functional or nutraceuticals.

## 3. Materials and Methods

### 3.1. Materials

All biomasses were collected in the southern part of Rio Grande do Sul, where the Pampa biome is located. The pampa biome is characterized by having a combination of temperate and subtropical climate, with well-defined seasons [7]. Sample collection was performed in October–December 2020 and analyzes in January–February 2021. The olive leaves (*Olea europaea* L., Arbequina) were provided by the Guarda Velha ranch (−31.50042, −53.51120), an olive oil producer located in Pinheiro Machado. The jaboticabas (*Plinia cauliflora*) and pecan nut shells (*Carya illinoinensis* K.Koch) were provided by a private rural property located in the Santa Flora district in Santa Maria (−29.88926, −53.87125). The araçás (*Psidium catteyanum* Sabine) were provided by private property in Candiota (−31.553710, −53.683664). All other reagents used were purchased from Sigma–Aldrich.

### 3.2. Sample Preparation and Extraction Procedure

First, the manual separation of the pecan nut endocarp, jaboticaba peel, and araçá peel were performed. Next, the raw materials related to the extracts were subjected to hygienization using 2–2.5% (*v*/*v*) sodium hypochlorite solution and washed in running water. After this step, each biomass was divided into two parts, the first part was convective-dried (Nova Ethics, 109–1) at 40 °C for 24 h [44,63,80], and the second part was stored in airtight bags at −18 °C for 24 h and then freeze-dried (Terroni-LS3000) for 48 h in high vacuum (≅0.3 mmHg) a—50 °C [81]. Finally, the samples were ground in an analytical mill (IKA, a11BS32) and then sent for sieving. In the next steps, particles with a diameter of less than 250 µm were used [26,44,63].

The biomasses were submitted to the extraction process of bioactive compounds by the solid–liquid maceration technique, adapted from Avila et al. [26], using a Dubnoff metabolic bath (SOLABSL-157/30) at 88 °C for 2 h. The extraction was performed at a ratio of 1:100 (ground biomass: solvent), in which two different solvents were used in the extraction of distilled water and 40% ethanolic solution (*v*/*v*) [32]. After extraction, the extracts were filtrated using a vacuum pump. In this work, four different extracts were obtained for each biomass. Therefore, all extractions will be carried out in duplicate.

### 3.3. Extracts Characterization

#### 3.3.1. Antioxidant Activity

Regarding the antioxidant activity of natural extracts, there is still no standardized method that provides the value of the antioxidant activity, but the scientific community accepts some methods. However, the chemical reactions in each method are different, and it is incorrect to state as “total antioxidant activity” the results obtained in different techniques, but rather to evaluate the chemical reaction in the method [82,83]. Furthermore, due to the different chemical reactions involved in these methods of analysis of antioxidant activity, the results of different techniques should not be compared [82,83,84].

The antioxidant potential of the obtained extracts was evaluated using the reagent 2,2-Diphenyl-1-picrylhydrazyl (DPPH) through the methodology proposed by Brand-Williams et al. [85] to determine the radical scavenging capacity of DPPH (RSC_DPPH_) and by the ferric reducing ability of plasma (FRAP) to determine the ferric reduction capacity (FRC).

RSC_DPPH_ was determined as follows: 200 µL of the natural extract was used for 7.8 mL of DPPH reagent, and then the samples were kept for 30 min in an environment without the presence of light for the reaction occurs. The reaction occurs due to the reduction in DPPH radicals that are captured by antioxidants present in the extracts. The results were determined using a UV-visible spectrophotometer (UV 755B, EQUILAM, Diadema, Brazil) at a wavelength of 517 nm. In addition, for control purposes, 200 µL of the extract was replaced in one sample with 200 µL of distilled water.

The FRAP methodology consists of preparing at least three dilutions of the extract to be analyzed. Then, in an environment without light, 270 μL of distilled water, 90 μL of each extract dilution, and 2.7 mL of the FRAP reagent should be added in Falcon tubes. The samples were then homogenized and kept at 37 °C for 30 min for the reaction. After this period, the mixtures were analyzed in a UV-Visible spectrophotometer (595 nm). In this methodology, a standard curve of ferrous sulfate is used to quantify the antioxidant activity, and the results obtained are expressed in µM Fe^+2^ g^−1^ [86].

#### 3.3.2. Determination of Total Phenolic Compounds

The total phenolic compounds (TP) were determined by the methodology described by Singleton and Rossi [87].

The TP was determined using 0.5 mL of extract, 10 mL of distilled water, and 1 mL of Folin Ciocalteu reagent. These were added in a test tube, and after 3 min, 8 mL of Na_2_CO_3_ solution (7.5% *w*/*v*) was added. Then, the samples were placed in an environment without light for 2 h. After this period, the samples were analyzed in a UV-Vis spectrophotometer (765 nm). The total phenolic compounds were calculated using a standard curve of gallic acid at different concentrations, and the results are expressed in mgGAE.g^−1^ (d.w.).

#### 3.3.3. Phenolic Compounds Analysis by High-Performance Liquid Chromatography—HPLC

The extracts were filtered through a 0.45 mm syringe filter in the HPLC analysis and transferred to vials. HPLC analyses were performed using an Agilent 64 (Agilent Technologies, Santa Clara, USA) equipped with a quaternary pump (1200 Series) and diode array detector (DAD) (Agilent 1260 Series Photo Diode Array Detector). The separation was conducted at 30 °C using a reversed-phase LC Column Eclipse Plus C18 (4.6 × 150 mm, 5 µm) (Supelco, Bellefonte, PA, USA). The column was eluted at a flow rate of 1 mL min^−1^, and the injection volume was 20 µL. Separation was achieved with a gradient of the solvent of 0.2% acetic acid (Solvent A), methanol (Solvent B), and acetonitrile (Solvent C), A/B/C, respectively. The gradient for the A/B/C start with 96/2/2%, followed by an 80/10/10% for the next 5 min; 70/15/15% for 5 min; 50/25/25% for 10 min; and back to the initial conditions with 96/2/2% for 10 min. Detection of phenolic compounds was performed at 280 nm and 520 nm. The phenolic compounds and anthocyanins were identified by comparison of their retention times of pure standards Sigma–Aldrich^®^ (Steineheim, Germany) and quantified using calibration curves.

#### 3.3.4. Microbiological Analysis

The natural extracts were evaluated for their inhibition and bactericidal potential against *Escherichia coli* (*E. coli,* ATCC 11229) and *Staphylococcus aureus* (*S*. *aureus,* ATCC 12598). The methodology used was macrodilution in broth, according to NCCLS [88]. All materials were previously sterilized in an autoclave (PRISMATEC-CS) at 120 °C for 15 min. For ethanol extracts, rotary evaporation was performed at 80 °C until the process was exhausted. Then, distilled water was added to the extracts to maintain the initial solution of 100 mL.

The bacterial culture of the microorganisms studied was carried out in nutrient broth (HIMEDIA) at 35 °C for 24 h in a bacteriological incubator (SOLAB, SL 101 After the incubation, the microorganism concentration was adjusted using the 0.5 McFarland scale (O. D. 0.08–0.1), using a UV-Vis spectrophotometer at a wavelength of 625 nm. After that, 0.5 mL of microorganism culture was added to test tubes containing 4.5 mL of extract at concentrations of 20, 40, 60, 80, and 90% (*v*/*v*). Dilutions were performed using previously sterilized Müller–Hinton broth. After preparation, all samples were incubated in a bacteriological incubator at 35 °C for 24 h. The results obtained were evaluated by visual analysis NCCLS [88]). The lowest concentration of extract at which there is no microbial growth is named as the minimum inhibitory concentration.

To verify the bactericidal potential of the extracts, the most diluted sample that showed inhibitory potential and the 2 subsequent ones were cultured in a Petri dish containing agar. Then, the samples were incubated at 35 °C for 24 h. After this period, microorganism growth in the agar was visually verified.

## 4. Conclusions

Select biomasses can be a rich source of phenolic compounds and free radical scavenging compounds. The phenolic compounds profile was correlated with the antioxidant and antimicrobial activities of the biomass extracts. The results obtained in this article described, for the first time, the extensive study of the parameters of solvent extract and drying technique and their impact on the final extract. The results reveal the powerful bioactive potential of four extracts from natural products, namely, olive leaves, jaboticaba peel, red araçá peel, and pecan nut shells. The prepared extracts showed high antioxidant activity and high total phenolic compounds.

Additionally, the most important phenolic compounds were quantified. Jaboticaba and araçá extracts present a substantial amount of cyanidin-3-glucoside and olive extract, an important amount of oleuropein. These phenolic compounds are possibly responsible for the antimicrobial activity of the extracts, which showed an important bactericidal effect against *Staphylococcus aureus* and *Escherichia coli* bacteria. Regarding the importance of the in vitro antimicrobial activity of the extracts, more studies are needed to corroborate the results through in vivo experiments. Nevertheless, these results exhibited promising results in the food, pharmaceutical, and cosmetics industries. The extracts can be used as natural additives for food preservation and coloring agents. Moreover, their bioactive potential can be explored as antioxidant and antimicrobial activity, being able to replace the use of synthetic chemical additives.

## Figures and Tables

**Table 1:** RSC_DPPH_ (%, d.w.) natural extracts results.

Extract	FW	FE	CW	CE
Jaboticaba peel	93.21 ± 0.07 ^a^	92.01 ± 0.73 ^a^	57.77 ± 0.56 ^b^	91.28 ± 0.29 ^a^
Olive leaf	95.23 ± 1.81 ^a^	93.05 ± 0.42 ^a^	91.89 ± 0.02 ^a^	94.85 ± 0.02 ^a^
Araçá peel	59.85 ± 1.49 ^c^	82.52 ± 1.37 ^a^	69.24 ± 0.23 ^b^	83.19 ± 0.61 ^a^
Pecan nut shell	74.79 ± 1.55 ^b^	90.32 ± 0.33 ^a^	92.26 ± 0.24 ^a^	91.66 ± 0.32 ^a^

The Tukey test (*p* < 0.05) was performed between samples of the same extract. Different letters in the same line indicate significant differences between samples.

**Table 2:** Ferric reduction capacity (µM Fe^+2^ g^−1^, d.w.) of the natural extracts.

Extract	FW	FE	CW	CE
Jaboticaba peel	1342.95 ± 2.73 ^b^	3525.69 ± 405.33 ^a^	1402.32 ± 144.62 ^b^	2111.17 ± 17.67 ^b^
Olive leaf	623.61 ± 37.01 ^ab^	484.14 ± 17.97 ^ab^	451.91 ± 3.72 ^b^	708.33 ± 71.12 ^a^
Araçá peel	128.21 ± 4.14 ^c^	222.53 ± 7.29 ^b^	171.98 ± 4.56 ^b^	424.19 ± 23.56 ^a^
Pecan nut shell	3449.46 ± 409.27 ^ab^	4266.53 ± 166.33 ^a^	2621.29 ± 100.68 ^b^	3979.07 ± 132.54 ^ab^

The Tukey test (*p* < 0.05) was performed between samples of the same extract. Different letters in the same line indicate significant differences between samples.

**Table 3:** Total phenolic compounds (mg_GAE_ g^−1^, d.w.) of the natural extracts.

Extract	FW	FE	CW	CE
Jaboticaba peel	88.02 ± 0.91 ^b^	122.63 ± 1.79 ^a^	46.84 ± 1.95 ^c^	81.47 ± 1.01 ^b^
Olive leaf	41.64 ± 0.65 ^c^	56.45 ± 0.91 ^b^	46.31 ± 1.04 ^c^	67.90 ± 1.42 ^a^
Araçá peel	17.52 ± 0.39 ^c^	30.61 ± 0.99 ^a^	13.94 ± 0.15 ^c^	25.63 ± 0.93 ^b^
Pecan nut shell	154.82 ± 5.67 ^b^	184.61 ± 1.69 ^a^	153.61 ± 1.07 ^b^	180.69 ± 2.56 ^a^

The Tukey test (*p* < 0.05) was performed between samples of the same extract. Different letters in the same line indicate significant differences between samples.

**Table 4:** Phenolic compounds (mg g^−1^, d. w.) of the natural extracts (mean ± SE, *n* = 4).

Phenolic Compounds	Extracts							
	Jaboticaba Peel		Olive Leaf		Pecan Nut Shell		Araçá Peel	
	Water	Ethanol	Water	Ethanol	Water	Ethanol	Water	Ethanol
Gallic acid	1.23 ± 0.04 ^a^	0.32 ± 0.01 ^d^	0.06 ± 0.00 ^f^	ND	0.75 ± 0.00 ^b^	0.22 ± 0.03 ^de^	0.16 ± 0.01 ^ef^	0.50 ± 0.04 ^c^
Caffeic acid	0.23 ± 0.02 ^b^	0.47 ± 0.02 ^a^	0.07 ± 0.00 ^c^	0.08 ± 0.00 ^c^	NI	NI	0.10 ± 0.00 ^c^	0.20 ± 0.00 ^b^
*p*-Coumaric acid	0.39 ± 0.01 ^b^	0.59 ± 0.05 ^a^	0.22 ± 0.00 ^c^	0.23 ± 0.00 ^c^	NI	NI	0.23 ± 0.00 ^c^	0.24 ± 0.00 ^c^
Chlorogenic acid	NI	NI	0.28 ± 0.00 ^b^	0.40 ± 0.04 ^a^	NI	NI	0.20 ± 0.00 ^b^	0.24 ± 0.01 ^b^
*trans*-Cinnamic acid	NQ	NQ	0.08 ± 0.00 ^b^	0.10 ± 0.00 ^a^	NI	NI	0.04 ± 0.00 ^c^	0.05 ± 0.00 ^c^
*trans*-Ferulic acid	NQ	NQ	0.28 ± 0.00 ^a^	0.22 ± 0.00 ^b^	NI	NI	NQ	NQ
Kaempferol	0.42 ± 0.00 ^c^	0.43 ± 0.00 ^c^	0.48 ± 0.00 ^b^	0.52 ± 0.01 ^a^	NI	NI	NQ	NQ
Quercetin	ND	ND	0.71 ± 0.00 ^b^	0.75 ± 0.02 ^a^	NI	NI	0.54 ± 0.00 ^d^	0.61 ± 0.00 ^c^
Cyanidin-3-glucoside	8.22 ± 0.34 ^a^	8.83 ± 0.70 ^a^	NI	NI	NI	NI	1.65 ± 0.00 ^b^	1.63 ± 0.00 ^b^
Hydroxytyrosol	NI	NI	3.40 ± 0.03 ^a^	3.71 ± 0.54 ^a^	NI	NI	NI	NI
Tyrosol	NI	NI	1.20 ± 0.00 ^a^	1.11 ± 0.23 ^a^	NI	NI	NI	NI
Oleuropein	NI	NI	66.81 ± 0.11 ^b^	130.45 ± 6.07 ^a^	NI	NI	NI	NI
Verbascoside	NI	NI	4.66 ± 0.07 ^b^	12.42 ± 0.61 ^a^	NI	NI	NI	NI
TPI	10.41 ± 0.41	10.64 ± 0.78	78.25 ± 0.21	149.99 ± 7.52	0.75 ± 00	0.22 ± 0.03	2.92 ± 0.01	3.47 ± 0.05

Mean values in each row with different letters are significantly different (ANOVA and Tukey test to compare many extracts or test t to compare two extracts, *p* < 0.05). TPI = total phenolic compounds identified. ND = below the limit of detection. NQ = below the limit of quantification. NI = not identified.

**Table 5:** Inhibition potential of natural extracts against microorganisms *Escherichia coli* and *Staphylococcus aureus*.

		Concentration of Extracts (%, *v*/*v*)				
Bacterium	Extracts	20	40	60	80	90
*Staphylococcus* *aureus*	Jaboticaba peel	NI	NI	I	I	I
	Olive leaf	NI	NI	I	I	I
	Pecan nut shell	-	-	-	-	-
	Araçá peel	NI	NI	NI	NI	I
*Escherichia* *coli*	Jaboticaba peel	NI	NI	I	I	I
	Olive leaf	NI	NI	I	I	I
	Pecan nut shell	-	-	-	-	-
	Araçá peel	NI	NI	NI	NI	I

I = inhibition identified. NI = no inhibition occurred.

## Data Availability

Not applicable.

## Supplementary Materials

The following supporting information can be downloaded at: https://www.mdpi.com/article/10.3390/molecules27206876/s1, Figure S1: HPLC chromatograms of ethanolic extracts of: jaboticaba peel (a,b), olive leaf (c,d), pecan nut shell (e,f) and araçá peel (g,h). Phenolic compounds: 1. Gallic acid, 2. Caffeic acid, 3. p-Coumaric acid, 4. Chlorogenic acid, 5. trans-Cinnamic acid, 6. trans-Ferulic acid, 7. Kaempferol, 8. Quercetin, 9. Cyanidin-3-glucoside, 10. Hydroxytyrosol, 11. Tyrosol, 12. Oleuropein and 13. Verbascoside.
