# Supplementary material for: Brazilian Agroindustrial Wastes as a Potential Resource of Bioative Compounds and Their Antimicrobial and Antioxidant Activities
Source: Molecules. 2022 Oct 13;27(20):6876. doi: 10.3390/molecules27206876 (PMC9606910; doi:10.3390/molecules27206876)

Figure S1.: HPLC chromatograms of ethanolic extracts of: jaboticaba peel (a,b), olive leaf (c,d), pecan nut shell (e,f) and araçá peel (g,h). Phenolic compounds: 1. Gallic acid, 2. Caffeic acid, 3. p-Coumaric acid, 4. Chlorogenic acid, 5. trans-Cinnamic acid, 6. trans-Ferulic acid, 7. Kaempferol, 8. Quercetin, 9. Cyanidin-3-glucoside, 10. Hydroxytyrosol, 11. Tyrosol, 12. Oleuropein and 13. Verbascoside.

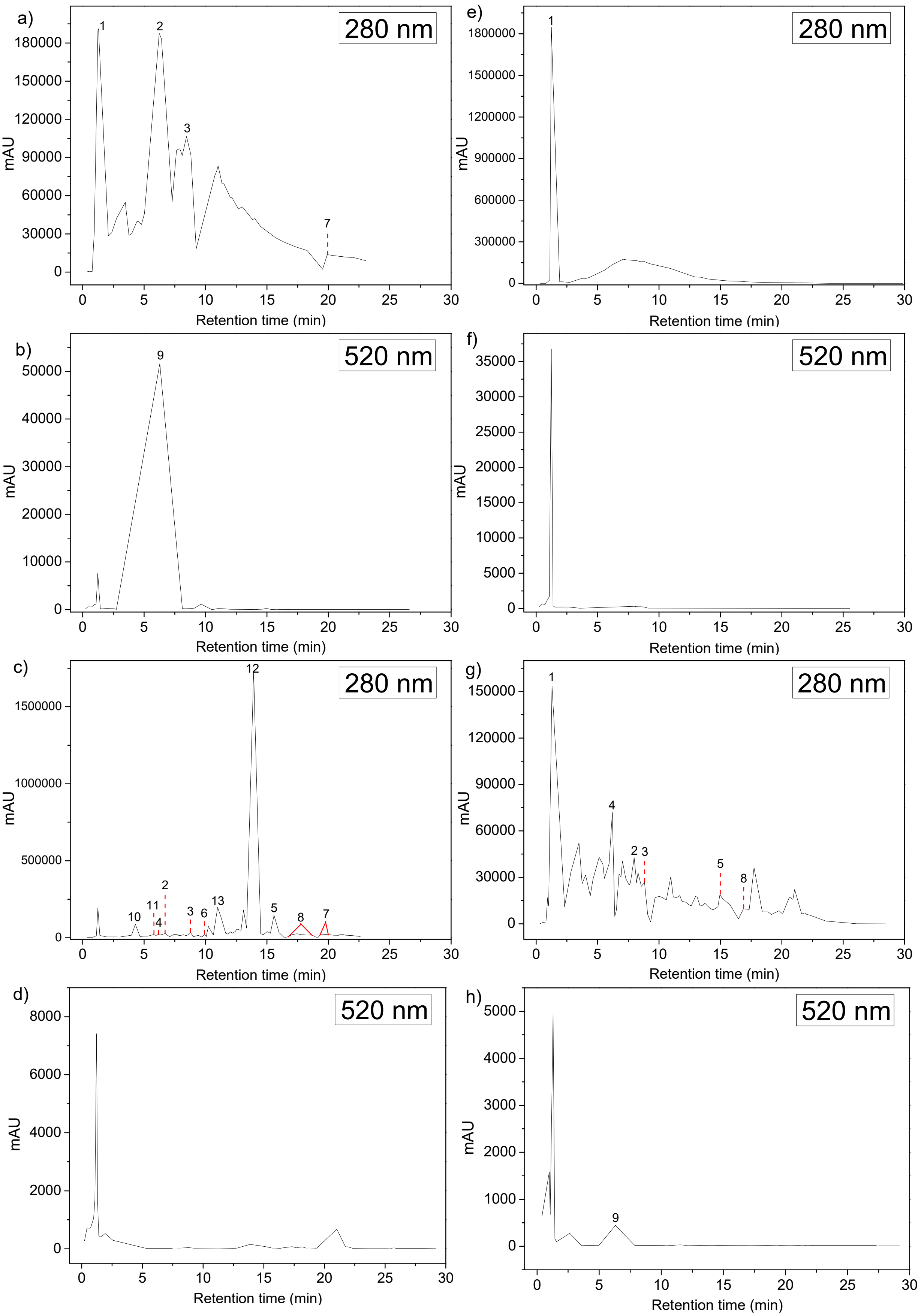

Supplement: Supplementary file 1 [file molecules-27-06876-s001.zip › molecules-1917511-supplementary.pdf]
